# In vivo translocator protein in females with autism spectrum disorder: a pilot study

**DOI:** 10.1038/s41386-024-01859-6

**Published:** 2024-04-13

**Authors:** Chieh-En Jane Tseng, Camila Canales, Rachel E. Marcus, Anjali J. Parmar, Baileigh G. Hightower, Jennifer E. Mullett, Meena M. Makary, Alison U. Tassone, Hannah K. Saro, Paige Hickey Townsend, Kirstin Birtwell, Lisa Nowinski, Robyn P. Thom, Michelle L. Palumbo, Christopher Keary, Ciprian Catana, Christopher J. McDougle, Jacob M. Hooker, Nicole R. Zürcher

**Affiliations:** 1 Athinoula A. Martinos Center for Biomedical Imaging, Massachusetts General Hospital, Charlestown, MA USA; 2 Harvard Medical School, Boston, MA USA; 3 Lurie Center for Autism, Massachusetts General Hospital, Lexington, MA USA; 4 Department of Pediatrics, Indiana University, Indianapolis, IN USA; 5 Systems and Biomedical Engineering Department, Faculty of Engineering, Cairo University, Cairo, Egypt

**Keywords:** Autism spectrum disorders, Neuroscience

## Abstract

Sex-based differences in the prevalence of autism spectrum disorder (ASD) are well-documented, with a male-to-female ratio of approximately 4:1. The clinical presentation of the core symptoms of ASD can also vary between sexes. Previously, positron emission tomography (PET) studies have identified alterations in the in vivo levels of translocator protein (TSPO)—a mitochondrial protein—in primarily or only male adults with ASD, with our group reporting lower TSPO relative to whole brain mean in males with ASD. However, whether in vivo TSPO levels are altered in females with ASD, specifically, is unknown. This is the first pilot study to measure in vivo TSPO in the brain in adult females with ASD using [^11^C]PBR28 PET-magnetic resonance imaging (MRI). Twelve adult females with ASD and 10 age- and TSPO genotype-matched controls (CON) completed one or two [^11^C]PBR28 PET–MRI scans. Females with ASD exhibited elevated [^11^C]PBR28 standardized uptake value ratio (SUVR) in the midcingulate cortex and splenium of the corpus callosum compared to CON. No brain area showed lower [^11^C]PBR28 SUVR in females with ASD compared to CON. Test-retest over several months showed stable [^11^C]PBR28 SUVR across time in both groups. Elevated regional [^11^C]PBR28 SUVR in females with ASD stand in stark contrast to our previous findings of lower regional [^11^C]PBR28 SUVR in males with ASD. Preliminary evidence of regionally elevated mitochondrial protein TSPO relative to whole brain mean in ASD females may reflect neuroimmuno-metabolic alterations specific to females with ASD.

## Introduction

Autism spectrum disorder (ASD) is characterized by difficulties in social communication and interaction and restrictive and repetitive patterns of behavior [1]. A striking observation regarding ASD is the significantly higher prevalence in males, with males diagnosed approximately four times more frequently than females [2]. In addition, the clinical presentation of the core symptoms of ASD can vary in males and females with ASD. Females with ASD may have fewer or less pronounced restricted interests and repetitive behaviors (reviewed in ref. [3]) and may demonstrate stronger social competence compared to males with ASD (reviewed in ref. [4]). The need to study both males and females with ASD has been recognized. Despite this, over 90% of neuroimaging studies in ASD in the last 20 years only studied males or did not study sex effects if females were included [5]. As a result, our understanding of neurobiology in females with ASD is particularly lacking.

Alterations in neuroimmune and mitochondrial mechanisms, both in the brain and periphery, have been repeatedly implicated in ASD (reviewed in refs. [6, 7]) [8–10]. Notably, naturally occurring sex differences have been observed in some of these neuroimmune and mitochondrial functions in humans [11, 12]. Post-mortem work showed that genes related to microglial and astrocyte function, which are upregulated in ASD, are more highly expressed in males compared to females [11]. Additionally, males have lower mitochondrial respiration than females in peripheral blood mononuclear cells [12], including in a segment of the electron transport chain (Complex I) that was found to be expressed at a lower level in post-mortem ASD brain tissue [13]. These sex differences in neuroimmune and mitochondrial processes demonstrate underlying biological differences in males and females and highlight the need to study these mechanisms in ASD in both sexes.

Translocator protein 18 kDa (TSPO), a mitochondrial protein which has neuroimmune functions [14], has previously been investigated in ASD [15–17]. Using positron emission tomography–magnetic resonance imaging (PET–MRI), we observed lower regional TSPO relative to the whole brain measured by standardized uptake value ratio (SUVR) in adult males with ASD (*N* = 15) compared to age-matched male controls [15]. Translocator protein levels were also assessed by another research group with [^18^F]FEPPA PET in a combined group of adult males and females with ASD, revealing either unchanged (*N* = 13, males = 8, females = 5) or lower (a subset of *N* = 11) TSPO levels in the brain using arterial blood-based PET quantification [17]. Another study which used the cerebellum of the controls as a reference region and the radiotracer [^11^C]PK11195, which has a lower ratio of specific to nonspecific binding than [^11^C]PBR28 [18], reported higher TSPO levels in males with ASD [17]. In parallel, Kato et al. recently reported lower in vivo mitochondrial Complex I in the brain in adult males with ASD [19]. Post-mortem studies have also revealed lower levels of mitochondrial proteins in adult males with ASD [9]. Taken together, this suggests a lower density of several mitochondrial proteins in males with ASD. Translocator protein levels have not been investigated specifically in females with ASD to date.

In this study, we used PET–MRI with a second-generation TSPO radiotracer, [^11^C]PBR28, to quantify in vivo TSPO in females with ASD. Given that individuals with ASD with comorbid intellectual disability are understudied, particularly in neuroimaging [20], we included females with ASD with a broad range of cognitive abilities. In terms of age, we focused on young adults as done previously for males [15]. We assessed whether (1) [^11^C]PBR28 SUVR are altered in females with ASD compared to female controls, (2) [^11^C]PBR28 SUVR are associated with ASD symptom severity, and (3) [^11^C]PBR28 SUVR in females with ASD and female controls are stable over several months. We hypothesize that females with ASD will have altered [^11^C]PBR28 SUVR compared to female controls and that [^11^C]PBR28 SUVR will be associated with symptom severity. Based on our previous findings in males, we expect [^11^C]PBR28 SUVR to be stable over several months.

## Methods and materials

### Study design

All participants were screened at the Lurie Center for Autism at Massachusetts General Hospital (MGH) and completed simultaneous PET–MRI scan(s) at the Athinoula A. Martinos Center for Biomedical Imaging. To meet inclusion criteria, participants had to be female, age 18–40 years, not smoke, and not be taking any anti-inflammatory drugs, immune-modulating drugs, or benzodiazepines other than lorazepam, desmethyldiazepam, and oxazepam, which are benzodiazepines that show negligible binding affinity to TSPO and are thought to not affect radiotracer binding in TSPO PET studies [21]. Participants had to have an intelligence quotient (IQ) at or above the range of moderate intellectual disability (IQ ≥ 35) for individuals with ASD and normal IQ (IQ ≥ 85) for controls (CON), which was measured using the abbreviated battery of the Stanford Binet Intelligence Scales, Fifth Edition (SB-5 ABIQ) [22] at the screening visit. Participants with ASD were excluded if they had a diagnosis of epilepsy and had a clinically significant seizure (one that required medical attention) or change in seizure medication in the last 6 months, as epilepsy can influence TSPO levels [23]. The rs6971 single-nucleotide polymorphism on exon 4 of the *TSPO* gene affects the binding affinity of [^11^C]PBR28 to TSPO [24]. Participants were, therefore, genotyped for the *TSPO* rs6971 polymorphism and only individuals with the C/C genotype (high affinity binders) and C/T genotype (mixed affinity binders) were scanned. Individuals with the T/T genotype, which confers negligible binding to [^11^C]PBR28 [24], were excluded. All participants were excluded if they were taking any illicit or recreational drugs (verified by the Discover Plus 12 Panel Dip Card urine drug test on the day of enrollment and the day of scan) or had any PET–MRI safety contraindications, including pregnancy and breastfeeding.

#### Demographic, medical history, and medication information

All participants included were of female sex. Participants provided demographic information which included gender, race, and ethnicity (Table 1). Information on medical history was collected by a physician (RPT, MP, CK, CJM) based on participant self-report. In addition, participants provided information on medication (current and over the past year) on the day of the scan.

**Table 1:** Demographic, intelligence, radiochemistry, behavioral, and current medication information.

	Scan 1			Scan 2		
	ASD (*N* = 12)	CON (*N* = 10)	*p* value	ASD (*N* = 4)	CON (*N* = 5)	*p* value
Demographics						
Age (years)	25.33 (5.88), Range: 19–37	25.70 (4.62), Range: 20–37	0.62	27.00 (3.92), Range: 22–31	27.60 (5.6), Range: 23–37	0.95
Sex	100% female	100% female		100% female	100% female	
Gender (F/M)	11/1	10/0	>0.99	4/0	5/0	>0.99
Race (White/Asian/Black)	11/1/0	7/2/1	0.36	4/0/0	3/1/1	0.36
Ethnicity (Hispanic/Non-Hispanic)	0/12	2/8	0.19	0/4	1/4	>0.99
TSPO genotype (C/C / C/T)	6/6	5/5	>0.99	2/2	3/2	>0.99
Body mass index	26.49 (5.03), Range: 18.90–35.70	23.2 (3.12), Range: 23.40–37.50	0.06	28.25 (6.58), Range: 19.60–27.80	25.82 (2.63), Range: 22.60–28.40	0.60
Intelligence						
SB-5 ABIQ	90.83 (25.36), Range: 50–124	101.80 (11.33), Range: 85–118	0.53	73.75 (17.73), Range: 61–100	98.80 (12.30), Range: 85–115	<0.05
Radiochemistry measures						
Injected dose (mCi)	14.15 (1.00), Range: 11.20–15.10	14.13 (0.86), Range: 11.80–14.83	0.91	14.51 (0.34), Range: 14.13–14.91	13.77 (1.25), Range: 11.95–14.82	0.51
Molar activity (mCi/nmol)	1.16 (0.21), Range: 0.77–1.49	1.49 (0.65), Range: 0.42–2.53	0.16	1.32 (0.21), Range: 1.13–1.57	1.36 (0.22), Range: 1.06–1.63	0.90
Injected mass (µg)	4.38 (0.93), Range: 3.24–6.57	4.25 (2.92), Range: 1.91–11.99	0.20	3.89 (0.66), Range: 3.12–4.60	3.62 (0.88), Range: 2.78–4.88	0.73
Behavioral measures						
ADOS-2 total score (social affect + restrictive and repetitive behavior)	16.00 (5.50), Range: 9–24, *N* = 11	N/A	N/A	16.25 (6.40), Range: 11–24	N/A	N/A
ADOS-2 social affect subscale score	12.18 (4.47), Range: 7–19, *N* = 11	N/A	N/A	12.25 (4.65), Range: 8–18	N/A	N/A
ADOS-2 restrictive and repetitive behavior subscale score	3.82 (1.66), Range: 1–6, *N* = 11	N/A	N/A	4.00 (1.83), Range: 2–6	N/A	N/A
ADI-R total score	40.5 (10.80), Range: 23–60, *N* = 11	N/A	N/A	48.50 (10.79), Range: 37–60	N/A	N/A
Current medication						
Atypical antipsychotics	3	0	N/A	0	0	N/A
Antidepressants	7	0	N/A	2	0	N/A
Anxiolytics	4	0	N/A	1	0	N/A
Anticonvulsants	3	0	N/A	1	0	N/A
Stimulants	2	0	N/A	0	0	N/A

*p* values were determined by Mann–Whitney *U* tests for all except sex, gender, race, and TSPO genotype for which a Fisher’s exact test was used. Values are reported as mean (standard deviation). The ADOS-2 social affect score ranges from 0 to 20, and the ADOS-2 restrictive and repetitive behavior score ranges from 0 to 10, with higher scores indicating more severe symptoms. The ADI-R total score is the sum of the social, communication, and restrictive, repetitive, and stereotyped behavior subscores.

*ASD* autism spectrum disorder, *CON* control, *N* number, *TSPO* translocator protein, *SB-5 ABIQ* Stanford Binet Intelligence Scales, Fifth Edition, abbreviated battery intelligence quotient, *ADOS-2* Autism Diagnostic Observation Schedule, Second Edition, *ADI-R* Autism Diagnostic Interview-Revised, *N/A* not applicable.

#### ASD diagnosis and clinical characterization

Individuals with ASD met the Diagnostic and Statistical Manual of Mental Disorders, 5th Edition (DSM-5) criteria for ASD [1] as assessed by a board-certified psychiatrist with experience in the diagnosis of ASD (RPT, MP, CK, CJM) at the MGH Lurie Center for Autism. The ASD diagnosis was corroborated by the Autism Diagnostic Interview-Revised (ADI-R) [25, 26] and the Autism Diagnostic Observation Schedule-2 (ADOS-2), Module 4 [27]. The ADOS-2 is a diagnostic tool which also provides metrics of ASD symptom severity in two domains, social affect and restrictive and repetitive behavior [28] (but see also ref. [29]). The sum of ADOS-2 social affect and restrictive and repetitive behavior subscale scores (ADOS-2 total score) was used to assess ASD symptom severity. The ADI-R and ADOS-2 were obtained for all except 1 participant.

### Study approval

The Institutional Review Board (IRB) of Mass General Brigham and the Radioactive Drug Research Committee (RDRC) approved the protocol for this study. Informed consent was provided by participants or their legally authorized representative (LAR; as appropriate for individuals with ASD and impaired capacity to provide consent). Capacity to consent was determined by a board-certified psychiatrist (RPT, MP, CK, CJM). Participants with ASD also provided written assent when LAR consent was obtained.

### PET–MRI data acquisition

All participants completed a scanner training protocol that included the option to view videos demonstrating procedures associated with a simultaneous PET–MRI scan at the Athinoula A. Martinos Center for Biomedical Imaging and included a training scan onsite, following our published protocol [30]. We have previously obtained high-quality data using this training protocol in individuals with ASD [15]. Participants were scanned using the Siemens BrainPET, a head-only PET camera inserted inside a 3 Tesla TIM Trio MR scanner and a head-only 8-channel receive MR radiofrequency coil. A multi-echo magnetization prepared rapid acquisition gradient echo (MEMPRAGE) T1-weighted structural scan with volumetric navigator-based prospective motion correction [31] was acquired (TR = 2530 ms, TE[1–4] = 1.66 ms, 3.53 ms, 5.4 ms, 7.27 ms, FOV = 280 mm, flip angle = 7 deg, voxel size = 1 mm isotropic). [^11^C]PBR28 PET was synthesized onsite by the Martinos Center radiopharmacy using methodology described previously [15]. A licensed nuclear medicine technologist administered ~15 mCi of [^11^C]PBR28 to participants as a slow bolus injection through an intravenous catheter in an arm or hand vein outside of the scanner (see Table 1).

### PET–MRI data analysis

The attenuation correction for PET data was conducted with the T1-weighted structural MRI using a statistical parametric mapping (SPM)-based, pseudo-computed tomography methodology [32, 33]. The PET data were reconstructed from prompt coincidences using the three-dimensional ordinary Poisson ordered-subset expectation maximization (3D OP-OSEM) algorithm with 1 subset and 32 iterations, corrected for normalization, isotope decay, dead time, photon attenuation, and random and scatter coincidences. The reconstructed standardized uptake value (SUV) image had a matrix size of 256 × 256 with 153 slices. The voxel size was 1.25 × 1.25 × 1.25 mm^3^.

The emission data collected from 60–90 min post-radioligand injection was reconstructed into six 5-min SUV images, re-aligned using MCFLIRT (FSL version 5.0.9, https://fsl.fmrib.ox.ac.uk/fsl) and averaged to generate each participant’s mean SUV image. The SUV image was linearly registered to the corresponding structural scan using spmregister (FreeSurfer version 5.3, https://surfer.nmr.mgh.harvard.edu). Skull-stripping was performed using a mask which included all the brain regions obtained from FreeSurfer’s automated parcellation and segmentation (version 6.0), based on the Desikan-Killiany atlas. A non-linear registration matrix was generated by registering the T1-weighted structural image to Montreal Neurological Institute (MNI) standard space using FSL’s FLIRT and FNIRT. This non-linear registration matrix was used to transform the skull-stripped SUV from subject space to MNI space. Each individual’s SUV image in MNI space was normalized by the whole brain mean excluding the ventricles (SUV ratio, SUVR) to account for individual differences in global signal. Whole brain mean SUV was not significantly different between ASD and CON groups (*p* = 0.87, Mann–Whitney *U* test). The SUVR images were spatially smoothed using a Gaussian filter with a full width at half maximum (FWHM) kernel size of 8 mm.

#### Long-term stability of [^11^C]PBR28 SUVR

For the participants who underwent a second [^11^C]PBR28 PET–MRI scan, which included 4 ASD and 5 CON, SUVR were assessed for 14 regions of interest (ROIs). These ROIs were previously used [15] and included the bilateral frontal, parietal, temporal and occipital lobes, and bilateral insula, cingulate, caudate, putamen, pallidum, thalamus, hippocampus/parahippocampal gyrus, amygdala, cerebellum based on the Automated Anatomical Labeling (AAL) human brain atlas [34]. A bilateral white matter ROI that was generated from the tissue probability maps in PMOD (PMOD Technologies LLC, Zurich, Switzerland), threshold = 98% probability was also included.

### Statistics

Whole brain voxelwise analyses using a general linear model were performed in FSL FEAT for the first scan. For group (ASD vs. CON) comparisons and associations with ADOS-2 total scores, linear regressions were conducted with mixed effects and ordinary least squares and a statistical threshold of *Z* > 2.3, *p*_cluster_ < 0.05 for cluster-based multiple comparison correction. Age and TSPO genotype were entered as variables of non-interest for all models. GraphPad Prism version 9.0 (GraphPad Software, La Jolla, CA) was used to conduct all other statistical tests, which included Mann–Whitney *U* tests for group comparison of continuous variables, Fisher’s exact tests for group comparison of categorical variables, and Pearson’s and Spearman’s correlations.

In order to assess long-term stability of [^11^C]PBR28 SUVR, Pearson’s *r* were calculated between the SUVR at these two time points in the 14 ROIs. In addition, Bland–Altman plots were created in Excel to assess the agreement between the two scans. The 95% limits of agreement were calculated as mean percent difference ±1.96 × standard deviation of the percent difference.

## Results

### Study participants

In total, 20 female participants with ASD and 14 typically developing female CON were enrolled in this study. Of these, 12 female participants with ASD and 10 age-matched CON completed a [^11^C]PBR28 PET–MRI scan. In addition, 4 individuals with ASD and 5 CON underwent a second [^11^C]PBR28 PET–MRI scan several months after the first scan in order to assess stability of [^11^C]PBR28 SUVR. The interval between the two scans was not significantly different between the two groups (ASD: 4.8 ± 0.5 months, CON: 4.5 ± 2.1 months, *p* > 0.99). Eight females with ASD who did not complete a scan either could not be included because they had the TSPO genotype T/T (*N* = 2), did not meet the DSM-5 criteria for ASD (*N* = 1), withdrew (*N* = 3), or were lost to follow-up (*N* = 2). Four CON who did not complete a scan were either ineligible due to not passing the urine drug test (*N* = 2) or due to a major physical illness (*N* = 1) or withdrew (*N* = 1). At the group-level, the ASD and CON participants did not differ in age, gender, race, ethnicity, TSPO genotype, body mass index, IQ, or radiochemistry measures of the radiotracer at the time of the first scan (Table 1).

### Whole brain voxelwise analysis comparing [^11^C]PBR28 SUVR between groups

**Fig. 1:**
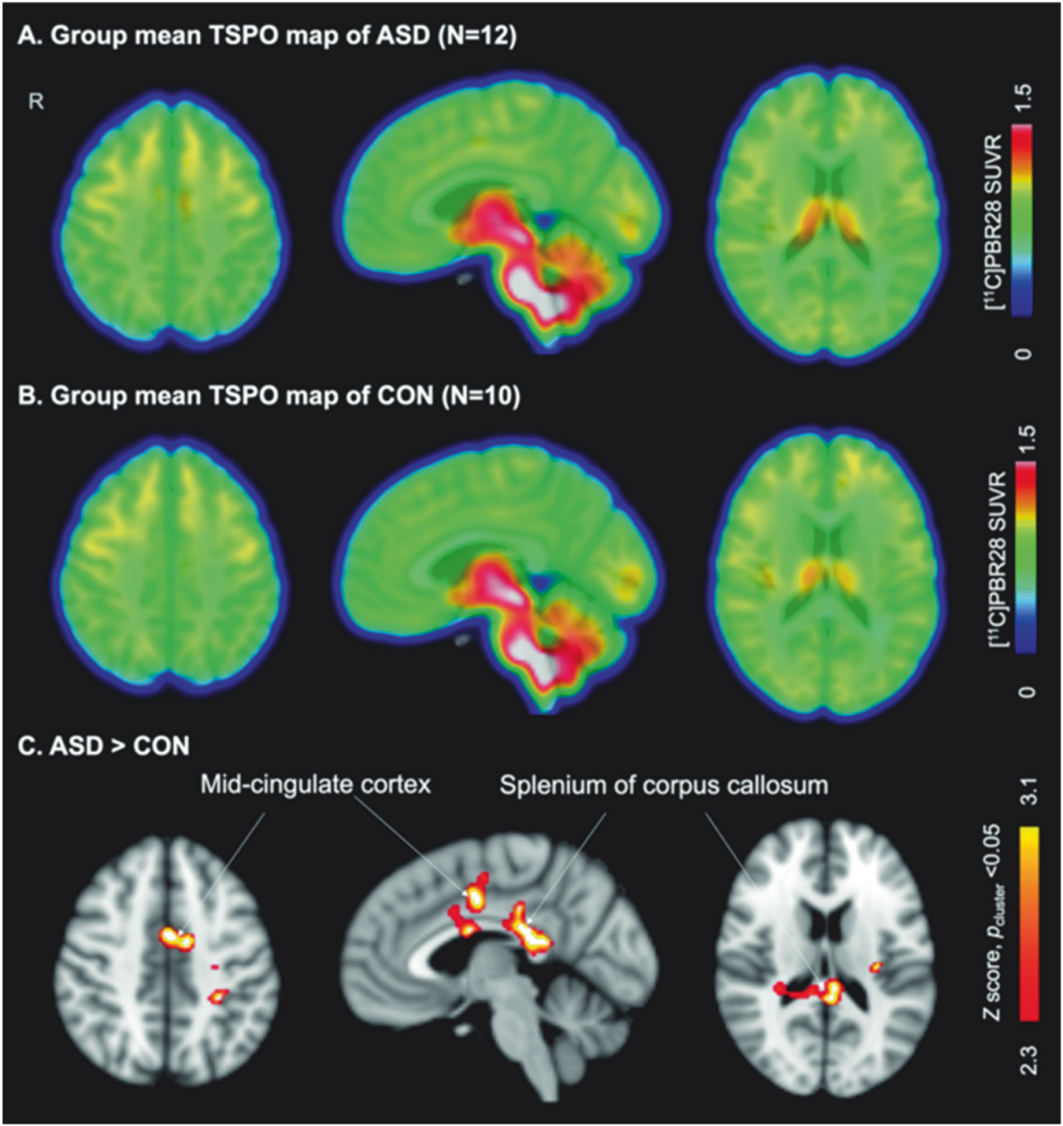
Elevated in vivo [^11^C]PBR28 SUVR in female adults with ASD compared to age- and TSPO genotype-matched CON. Group mean [^11^C]PBR28 SUVR maps of females with ASD (top) and female CON (middle). Statistical map from voxelwise comparison of [^11^C]PBR28 SUVR between groups, controlled for age and TSPO genotype, shows elevated regional TSPO levels relative to whole brain mean in the midcingulate cortex and splenium of the corpus callosum in ASD (*N* = 12) compared to CON (*N* = 10) (*Z* > 2.3, *p*_cluster_ < 0.05) (bottom). TSPO translocator protein, ASD autism spectrum disorder, CON control, SUVR standardized uptake value ratio, *N* number.

Elevated [^11^C]PBR28 SUVR were found in the midcingulate cortex (MCC) and splenium of the corpus callosum in female adults with ASD compared to age- and TSPO genotype-matched CON (*Z* > 2.3, *p*_cluster_ < 0.05, Fig. 1). The cluster with the MCC was 7064 mm^3^ and the cluster with the splenium was 11,016 mm^3^. For visualization purposes, individual data points for the [^11^C]PBR28 SUVR residuals (controlling for age and TSPO genotype) in the posthoc clusters are shown in Supplementary Fig. S1. No brain region showed lower [^11^C]PBR28 SUVR in ASD compared to CON.

Using the simultaneously collected MRI data, the structural volume of the posthoc clusters where group differences in [^11^C]PBR28 SUVR were found were assessed. No structural volume differences were found in these areas in ASD compared to CON (see Supplementary).

Exploratory analysis comparing [^11^C]PBR28 SUVR in the posthoc clusters in ASD with IQ ≥ 85 compared to ASD with IQ < 85 showed no difference between these two subgroups in either cluster (see Supplementary and Fig. S2). One subject with ASD had a history of epilepsy and one subject with ASD was taking lorazepam which is a benzodiazepine with negligible binding to TSPO [21]. Analysis without either of these subjects did not significantly change the findings (see Supplementary Figs. S3, S4).

### Association between [^11^C]PBR28 SUVR and ASD symptom severity

**Fig. 2:**
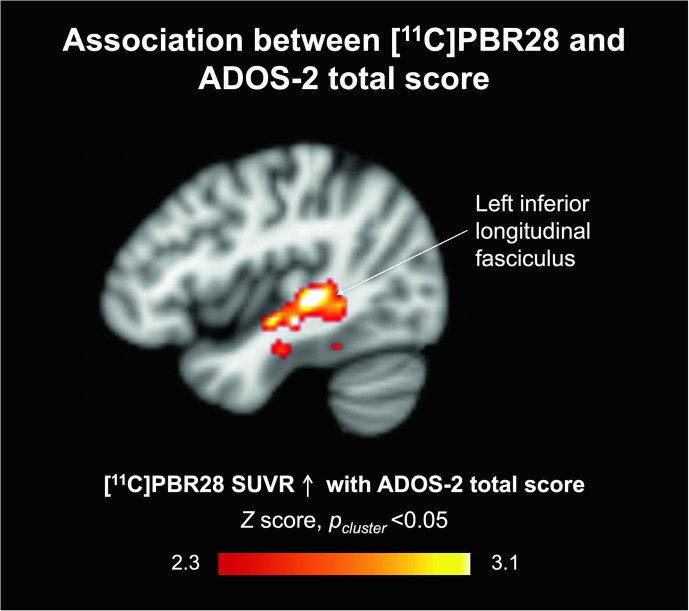
Regional TSPO levels relative to whole brain mean in the left inferior longitudinal fasciculus were positively correlated with ASD symptom severity measured by ADOS-2 total scores in females with ASD (*Z* > 2.3, *p*_cluster_ < 0.05). TSPO translocator protein, ASD autism spectrum disorder.

The ADOS-2 total scores were positively associated with [^11^C]PBR28 SUVR in the left inferior longitudinal fasciculus (ILF) (*Z* > 2.3, *p*_cluster_ < 0.05, Fig. 2). Individual data points for [^11^C]PBR28 SUVR residuals (controlling for age and TSPO genotype) in the posthoc cluster plotted against ADOS-2 total scores are shown in Supplementary Fig. S5 for visualization purposes. There was no region where ADOS-2 total scores were negatively associated with [^11^C]PBR28 SUVR. The [^11^C]PBR28 SUVR in the posthoc clusters where group differences were found were not associated with the ADOS-2 total score, controlling for age and TSPO genotype (MCC cluster: Spearman’s *r* = 0.33, *p* = 0.32; splenium cluster: Spearman’s *r* = 0.009, *p* = 0.99).

### [^11^C]PBR28 SUVR stability

**Fig. 3:**
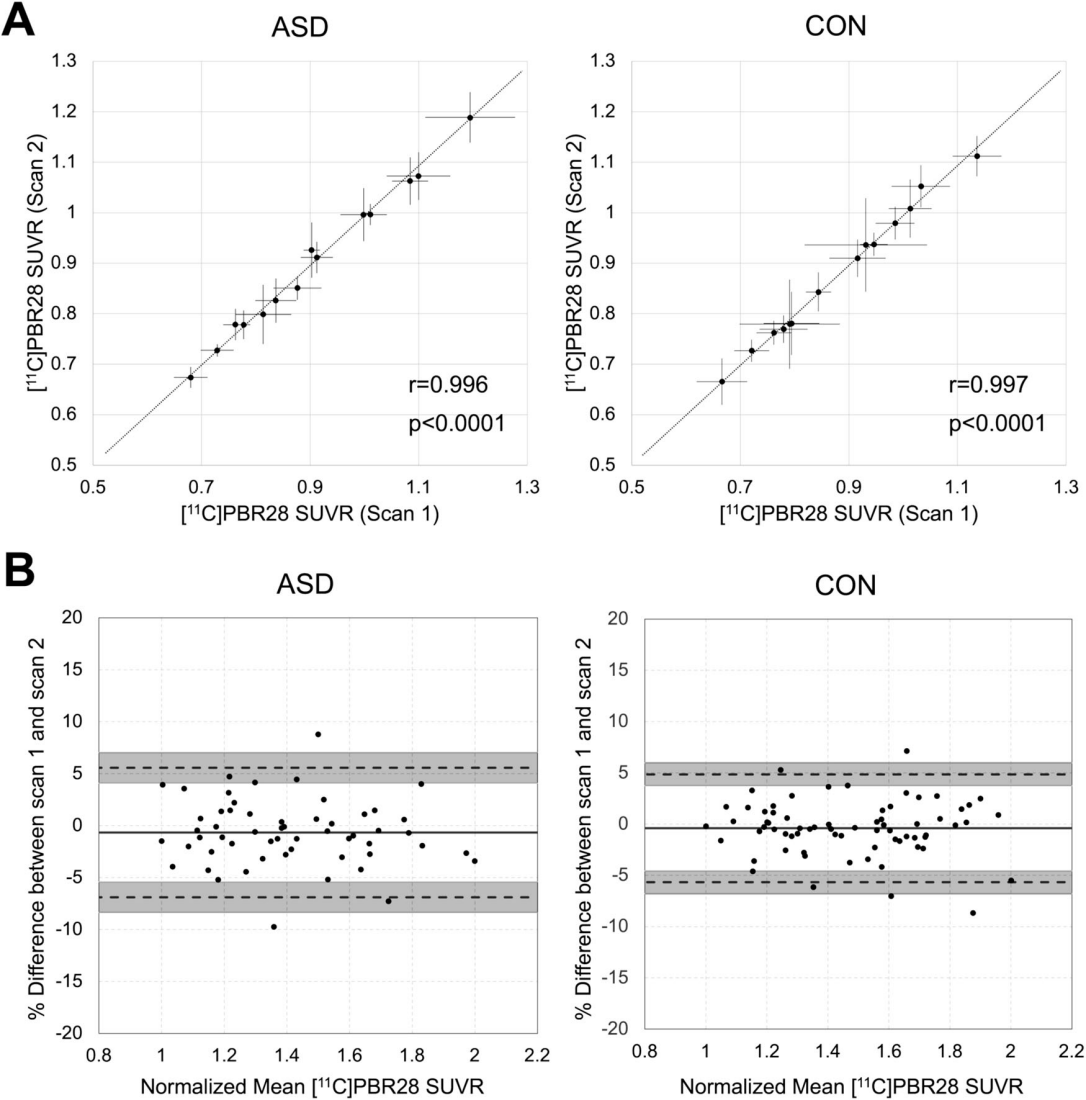
Stable [^11^C]PBR28 SUVR in ASD and CON. **A** Correlation plots show that [^11^C]PBR28 SUVR over a mean rescan interval of 4.6 months are highly correlated in ASD (*N* = 4) and CON (*N* = 5). Each dot represents the mean [^11^C]PBR28 SUVR in a brain region and the lines indicate the standard deviation. The dotted line represents the identity line. **B** Bland–Altman plots of between-scan agreement. Mean [^11^C]PBR28 SUVR values were normalized to a range of 1–2 in ASD and CON. The solid line represents the mean percent difference between scan 1 and scan 2, and points represent each ROI from each participant. The upper and lower limits of agreement are depicted with dotted lines and the shaded regions are the 95% confidence interval of the upper and lower limits of agreement. SUVR standardized uptake value ratio, ASD autism spectrum disorder, CON control, *N* number, ROI region of interest.

[^11^C]PBR28 SUVR was stable over around a 4.6-month period in ASD and CON, with scan 1 and scan 2 being strongly correlated (ASD: Pearson’s *r* = 0.996, *p* < 0.0001; CON: Pearson’s *r* = 0.997, *p* < 0.0001; Fig. 3A). High scan-rescan agreement was found in both ASD and CON with the bias of almost all regions falling within the 95% limits of agreement. The mean bias was −0.66 ± 3.17% for ASD and −0.40 ± 2.68% for CON (Fig. 3B). [^11^C]PBR28 SUVR values in each ROI are presented in Supplementary Table S1. The ASD and CON participants that completed a second scan did not differ in age, TSPO genotype, body mass index, or radiochemistry measures of the radiotracer. The participants with ASD who completed a second scan had lower IQ than the CON participants who completed a second scan (Table 1).

## Discussion

This study, the first TSPO imaging study to focus specifically on females with ASD, presents preliminary evidence of elevated [^11^C]PBR28 SUVR in the MCC and splenium of the corpus callosum in female young adults with ASD compared to age- and TSPO genotype-matched female CON. The regional elevations in TSPO (compared to whole brain mean) in female adults with ASD stand in contrast with the regional reductions in TSPO (compared to whole brain mean) in male adults with ASD compared to male controls using the same radiotracer and analysis methodology [15]. In addition, we found that [^11^C]PBR28 SUVR was stable over several months.

While our approach allows us to assess [^11^C]PBR28 uptake, generally thought of as correlated to the availability of TSPO, the neurobiological interpretation of any alterations seen is more complex and cannot be fully defined in this study. For example, regional TSPO alterations may reflect altered density of TSPO expressing cells and recent work suggested that under pro-inflammatory conditions, TSPO elevations may be associated with increases in inflammatory cell density in humans [35]. Numerous functions have been associated with changes in TSPO, including mitochondrial function (reviewed in ref. [36]) and neuronal activity [37]. The elevated [^11^C]PBR28 SUVR found in this study in females with ASD may not reflect the same biological processes as in other disorders [38] that show high TSPO levels, including neurodegenerative disorders (reviewed in ref. [39]). Elevated [^11^C]PBR28 SUVR in females with ASD may reflect neuroimmune alterations that have been previously reported in ASD (reviewed in ref. [40]). Translocator protein overexpression has also been associated with anti-inflammatory effects (reviewed in ref. [36]) and preclinical work has shown higher expression of anti-inflammatory markers in females compared to males in an early-life immune activation mouse model that leads to autism-like symptoms [41]. To pinpoint the biological process associated with elevated [^11^C]PBR28 SUVR in ASD females, it will be critical to identify the cell type(s) driving this difference, as TSPO is expressed in numerous cells including microglia, astrocytes, neurons, and endothelial cells [14, 37]. Despite the complexity of the interpretation, in vivo imaging of TSPO provides clues to which brain regions to investigate and gives us the opportunity to elucidate condition-related mechanisms in the future.

In terms of implicated brain regions, previous MRI studies have reported alterations in the regions where elevated TSPO was observed in this study, the MCC and splenium of the corpus callosum, in ASD [42–44]. The MCC is involved in reward-based decision-making and social behavior (reviewed in ref. [45]). Alterations in the structure and function of the corpus callosum have been reported in ASD (reviewed in ref. [46]), including white matter deficits in the splenium [43, 44]. In our sample, we did not find differences in structural volume in the areas where group differences in [^11^C]PBR28 SUVR were found (i.e., MCC and splenium).

ADOS-2 total scores were positively associated with [^11^C]PBR28 SUVR in the left ILF, indicating that higher regional TSPO in the left ILF is associated with greater ASD symptom severity. This association is different from what was previously observed in males with ASD where lower TSPO levels tended to be associated with greater ASD symptom severity as measured by the ADOS-2 total score [15]. A larger sample size to confirm this preliminary observation is warranted. The ILF is a white matter tract that connects the occipital lobe and anterior temporal brain regions and is involved in visual, face, and emotional processing (reviewed in ref. [47]), processes that are altered in ASD (reviewed in ref. [48]). The ILF shows lower white matter integrity in adolescents with ASD [49, 50]. Weaker white matter integrity of the ILF is also associated with lower social skills in adolescents and adults with ASD [49]. Whether higher TSPO levels co-occur with white matter alterations in ASD remains to be investigated.

It is notable that the direction of difference in females was higher TSPO rather than lower as we previously observed in males [15] and others have reported in a mixed group of males and females with ASD [17]. Sex-specific alterations in regional TSPO levels may relate to differing neurobiology of ASD in females and males. Whether changes in regional TSPO levels arise from the same process or cell source in males and females with ASD will need to be identified. Numerous preclinical autism models associated with immune mechanisms have shown sex-specific effects [41, 51, 52]. It is worth noting that higher in vivo TSPO levels have been previously reported in patients with active episodes of major depressive disorder (reviewed in ref. [53]). Anecdotally in this study, 5 out of 12 females with ASD self-reported a history of co-occurring depression (compared to none of the males with ASD in our previous study [15]). The [^11^C]PBR28 SUVR in the posthoc clusters where group differences were found were not different between those females with ASD who did and did not self-report a history of depression (see Supplementary). However, we acknowledge that this current study did not assess current burden of depressive symptoms, which represents a limitation. Future work could assess the relationship between TSPO and depressive symptoms in females with ASD.

While the sample size of this study is not large (*N* = 12, with *N* = 19 enrolled) and our findings therefore have to be considered preliminary, it is to our knowledge, the largest sample of females with ASD among PET studies in ASD that included females as a separate group or assessed sex effects (see *N* = 4 [54, 55], *N* = 6 [56], and *N* = 11 [57, 58]). This study adds to the scarce knowledge that we have regarding molecular markers in females with ASD, as to the best of our knowledge, only 5 out of over 70 PET studies in ASD to date have assessed females with ASD either as a separate group or assessed group-by-sex interaction effects. Specifically, this is the first TSPO PET–MRI study conducted exclusively in females with ASD. Recruitment of females with ASD for [^11^C]PBR28 PET–MRI studies is particularly challenging because of the lower prevalence of ASD in females in addition to general constraints for [^11^C]PBR28 PET–MRI studies, including the inability to include individuals with the TSPO genotype that confers low binding affinity, PET–MRI safety-contraindications, and challenges associated with neuroimaging studies in general and with studies involving radiation. It is notable that TSPO PET studies of similar sample size have led to disease-specific observations (e.g., schizophrenia: *N* = 12 [59], ASD: *N* = 11 [17]). As in most ASD research, we must acknowledge potential confounding effects due to medications, but our study did exclude for those benzodiazepines that were considered a confounding factor for [^11^C]PBR28 imaging. Furthermore, we opted to not perform arterial line procedures due to the invasive nature of these procedures, particularly given the inclusion of individuals with impaired decision-making. We acknowledge the limitations associated with our use of [^11^C]PBR28 SUVR with whole brain normalization. [^11^C]PBR28 PET does not have a true reference region for ratio metrics. Prior work has found high correlation between [^11^C]PBR28 SUVR and distribution volume ratios in several disorders using different pseudo-reference regions [60–62]. To date, a pseudo-reference region for [^11^C]PBR28 has not been determined in ASD, which would be required, absent arterial input function, to understand any global changes [63]. Previous work has demonstrated that when using the whole brain as a reference region, focal differences in [^11^C]PBR28 SUVR that match disease-specific pathological abnormalities observed through post-mortem analysis can be detected [64, 65]. The fundamental assumption of using whole brain normalization, with the prerequisite of no group differences in whole brain SUV (as was the case in this study), is that the emphasis is on regional effects and that global changes in signal may not represent underlying pathophysiology. However, we acknowledge that using whole brain normalization deviates from traditional SUVR approaches with ideal reference regions and comes with the complexity that the brain areas being assessed are included in the region used for normalization. Another potential limitation is that we have not performed partial volume effect correction. However, the BrainPET prototype’s spatial resolution is ~2.5 mm FWHM at the center of the field of view [66], which is better than that of whole-body PET devices. Furthermore, we have previously carefully characterized its spatially variant point spread function [67] and this information was included in the image reconstruction used here.

The findings in this study, in conjunction with the findings from our previous TSPO PET–MR imaging study in males with ASD [15], provide preliminary evidence that the in vivo TSPO profile of females and males with ASD is different, with elevated regional TSPO in females with ASD and lower regional TSPO in males with ASD compared to sex-matched CON. Whether this contributes to sex differences in ASD prevalence and/or presentations of the core symptoms of ASD in individuals with ASD will need to be further elucidated.

### Supplementary information

Supplement

## Data Availability

Custom codes used in this study will be made available upon reasonable request.

## References

[1] American Psychiatric Association. Diagnostic and statistical manual of mental disorders. 5th ed. Arlington, VA: American Psychiatric Association; 2013.

[2] Maenner MJ, Warren Z, Williams AR, Amoakohene E, Bakian AV, Bilder DA (2023). Prevalence and characteristics of autism spectrum disorder among children aged 8 years—autism and developmental disabilities monitoring network, 11 sites, United States, 2020. MMWR Surveill Summ.

[3] Bourson L, Prevost C. Characteristics of restricted interests in girls with ASD compared to boys: a systematic review of the literature. Eur Child Adolesc Psychiatry. 2022. 10.1007/s00787-022-01998-5 10.1007/s00787-022-01998-5 35644857

[4] Lai MC, Lombardo MV, Auyeung B, Chakrabarti B, Baron-Cohen S (2015). Sex/gender differences and autism: setting the scene for future research. J Am Acad Child Adolesc Psychiatry.

[5] Mo K, Sadoway T, Bonato S, Ameis SH, Anagnostou E, Lerch JP (2021). Sex/gender differences in the human autistic brains: a systematic review of 20 years of neuroimaging research. Neuroimage Clin.

[6] Rossignol DA, Frye RE (2012). Mitochondrial dysfunction in autism spectrum disorders: a systematic review and meta-analysis. Mol Psychiatry.

[7] Masi A, Quintana DS, Glozier N, Lloyd AR, Hickie IB, Guastella AJ (2015). Cytokine aberrations in autism spectrum disorder: a systematic review and meta-analysis. Mol Psychiatry.

[8] Al-Haddad BJS, Jacobsson B, Chabra S, Modzelewska D, Olson EM, Bernier R (2019). Long-term risk of neuropsychiatric disease after exposure to infection in utero. JAMA Psychiatry.

[9] Tang G, Gutierrez Rios P, Kuo SH, Akman HO, Rosoklija G, Tanji K (2013). Mitochondrial abnormalities in temporal lobe of autistic brain. Neurobiol Dis.

[10] Gupta S, Ellis SE, Ashar FN, Moes A, Bader JS, Zhan J (2014). Transcriptome analysis reveals dysregulation of innate immune response genes and neuronal activity-dependent genes in autism. Nat Commun.

[11] Werling DM, Parikshak NN, Geschwind DH (2016). Gene expression in human brain implicates sexually dimorphic pathways in autism spectrum disorders. Nat Commun.

[12] Silaidos C, Pilatus U, Grewal R, Matura S, Lienerth B, Pantel J (2018). Sex-associated differences in mitochondrial function in human peripheral blood mononuclear cells (PBMCs) and brain. Biol Sex Differ.

[13] Anitha A, Nakamura K, Thanseem I, Matsuzaki H, Miyachi T, Tsujii M (2013). Downregulation of the expression of mitochondrial electron transport complex genes in autism brains. Brain Pathol.

[14] Notter T, Coughlin JM, Gschwind T, Weber-Stadlbauer U, Wang Y, Kassiou M (2018). Translational evaluation of translocator protein as a marker of neuroinflammation in schizophrenia. Mol Psychiatry.

[15] Zürcher NR, Loggia ML, Mullett JE, Tseng C, Bhanot A, Richey L (2021). [(11)C]PBR28 MR–PET imaging reveals lower regional brain expression of translocator protein (TSPO) in young adult males with autism spectrum disorder. Mol Psychiatry.

[16] Suzuki K, Sugihara G, Ouchi Y, Nakamura K, Futatsubashi M, Takebayashi K (2013). Microglial activation in young adults with autism spectrum disorder. JAMA Psychiatry.

[17] Simpson D, Gharehgazlou A, Da Silva T, Labrie-Cleary C, Wilson AA, Meyer JH (2022). In vivo imaging translocator protein (TSPO) in autism spectrum disorder. Neuropsychopharmacology.

[18] Kreisl WC, Fujita M, Fujimura Y, Kimura N, Jenko KJ, Kannan P (2010). Comparison of [(11)C]-(R)-PK 11195 and [(11)C]PBR28, two radioligands for translocator protein (18 kDa) in human and monkey: implications for positron emission tomographic imaging of this inflammation biomarker. Neuroimage.

[19] Kato Y, Yokokura M, Iwabuchi T, Murayama C, Harada T, Goto T, et al. Lower availability of mitochondrial complex I in anterior cingulate cortex in autism: a positron emission tomography study. Am J Psychiatry. 2023;180:277–284. 10.1176/appi.ajp.22010014 36069020

[20] Jack A, Pelphrey KA (2017). Annual Research Review: understudied populations within the autism spectrum – current trends and future directions in neuroimaging research. J Child Psychol Psychiatry.

[21] Kalk NJ, Owen DR, Tyacke RJ, Reynolds R, Rabiner EA, Lingford-Hughes AR (2013). Are prescribed benzodiazepines likely to affect the availability of the 18 kDa translocator protein (TSPO) in PET studies?. Synapse.

[22] Roid G. Stanford-Binet Intelligence Scales. 5th ed. Itasca, IL: Riverside Publishing; 2003.

[23] Gershen LD, Zanotti-Fregonara P, Dustin IH, Liow J-S, Hirvonen J, Kreisl WC (2015). Neuroinflammation in temporal lobe epilepsy measured using positron emission tomographic imaging of translocator protein. JAMA Neurol.

[24] Owen DR, Yeo AJ, Gunn RN, Song K, Wadsworth G, Lewis A (2012). An 18-kDa translocator protein (TSPO) polymorphism explains differences in binding affinity of the PET radioligand PBR28. J Cereb Blood Flow Metab.

[25] Lord C, Rutter M, Le Couteur A (1994). AutIsm Diagnostic Interview – Revised: a revised version of a diagnostic interview for caregivers of individuals with possible pervasive developmental disorders. J Autism Dev Disord.

[26] Rutter M, Le Couteur A, Lord C. Autism Diagnostic Interview – Revised. Los Angeles, CA: Western Psychological Services; 2003.

[27] Lord C, Rutter M, DiLavore PC, Risi S, Gotham K, Bishop S. Autism Diagnostic Observation Schedule, 2nd ed. (ADOS-2). Los Angeles, CA: Western Psychological Services; 2012.

[28] Hus V, Lord C (2014). The autism diagnostic observation schedule, module 4: revised algorithm and standardized severity scores. J Autism Dev Disord.

[29] Morrier MJ, Ousley OY, Caceres-Gamundi GA, Segall MJ, Cubells JF, Young LJ (2017). Brief report: relationship between ADOS-2, module 4 calibrated severity scores (CSS) and social and non-social standardized assessment measures in adult males with autism spectrum disorder (ASD). J Autism Dev Disord.

[30] Smith CJ, Bhanot A, Norman E, Mullett JE, Bilbo SD, McDougle CJ (2019). A protocol for sedation free MRI and PET imaging in adults with autism spectrum disorder. J Autism Dev Disord.

[31] Tisdall MD, Reuter M, Qureshi A, Buckner RL, Fischl B, van der Kouwe AJW (2016). Prospective motion correction with volumetric navigators (vNavs) reduces the bias and variance in brain morphometry induced by subject motion. Neuroimage.

[32] Izquierdo-Garcia D, Hansen AE, Forster S, Benoit D, Schachoff S, Furst S (2014). An SPM8-based approach for attenuation correction combining segmentation and nonrigid template formation: application to simultaneous PET/MR brain imaging. J Nucl Med.

[33] Ladefoged CN, Law I, Anazodo U, St Lawrence K, Izquierdo-Garcia D, Catana C (2017). A multi-centre evaluation of eleven clinically feasible brain PET/MRI attenuation correction techniques using a large cohort of patients. Neuroimage..

[34] Tzourio-Mazoyer N, Landeau B, Papathanassiou D, Crivello F, Etard O, Delcroix N (2002). Automated anatomical labeling of activations in SPM using a macroscopic anatomical parcellation of the MNI MRI single-subject brain. Neuroimage.

[35] Nutma E, Fancy N, Weinert M, Tsartsalis S, Marzin MC, Muirhead RCJ (2023). Translocator protein is a marker of activated microglia in rodent models but not human neurodegenerative diseases. Nat Commun.

[36] Notter T, Coughlin JM, Sawa A, Meyer U (2018). Reconceptualization of translocator protein as a biomarker of neuroinflammation in psychiatry. Mol Psychiatry.

[37] Notter T, Schalbetter SM, Clifton NE, Mattei D, Richetto J, Thomas K (2021). Neuronal activity increases translocator protein (TSPO) levels. Mol Psychiatry.

[38] Liu GJ, Middleton RJ, Hatty CR, Kam WW, Chan R, Pham T (2014). The 18 kDa translocator protein, microglia and neuroinflammation. Brain Pathol.

[39] Bradburn S, Murgatroyd C, Ray N (2019). Neuroinflammation in mild cognitive impairment and Alzheimer’s disease: a meta-analysis. Ageing Res Rev.

[40] McDougle C, Landino S, Vahabzadeh A, O’Rourke J, Zürcher N, Finger B, et al. Toward an immune-mediated subtype of autism spectrum disorder. Brain Res. 2014;1617:72–92. 10.1016/j.brainres.2014.09.048 25445995

[41] Carlezon WA, Kim W, Missig G, Finger BC, Landino SM, Alexander AJ (2019). Maternal and early postnatal immune activation produce sex-specific effects on autism-like behaviors and neuroimmune function in mice. Sci Rep.

[42] Uppal N, Wicinski B, Buxbaum JD, Heinsen H, Schmitz C, Hof PR (2014). Neuropathology of the anterior midcingulate cortex in young children with autism. J Neuropathol Exp Neurol.

[43] Sui YV, Donaldson J, Miles L, Babb JS, Castellanos FX, Lazar M (2018). Diffusional kurtosis imaging of the corpus callosum in autism. Mol Autism.

[44] Hattori A, Kamagata K, Kirino E, Andica C, Tanaka S, Hagiwara A (2019). White matter alterations in adult with autism spectrum disorder evaluated using diffusion kurtosis imaging. Neuroradiology.

[45] Apps M, Lockwood P, Balsters J (2013). The role of the midcingulate cortex in monitoring others’ decisions. Front Neurosci.

[46] Valenti M, Pino MC, Mazza M, Panzarino G, Di Paolantonio C, Verrotti A (2020). Abnormal structural and functional connectivity of the corpus callosum in autism spectrum disorders: a review. Rev J Autism Dev Disord.

[47] Herbet G, Zemmoura I, Duffau H (2018). Functional anatomy of the inferior longitudinal fasciculus: from historical reports to current hypotheses. Front Neuroanat.

[48] Yeung MK (2022). A systematic review and meta-analysis of facial emotion recognition in autism spectrum disorder: the specificity of deficits and the role of task characteristics. Neurosci Biobehav Rev.

[49] Bakhtiari R, Zürcher NR, Rogier O, Russo B, Hippolyte L, Granziera C (2012). Differences in white matter reflect atypical developmental trajectory in autism: a tract-based spatial statistics study. Neuroimage Clin.

[50] Boets B, Van Eylen L, Sitek K, Moors P, Noens I, Steyaert J (2018). Alterations in the inferior longitudinal fasciculus in autism and associations with visual processing: a diffusion-weighted MRI study. Mol Autism.

[51] Missig G, Robbins JO, Mokler EL, McCullough KM, Bilbo SD, McDougle CJ (2020). Sex-dependent neurobiological features of prenatal immune activation via TLR7. Mol Psychiatry.

[52] Bolton JL, Marinero S, Hassanzadeh T, Natesan D, Le D, Belliveau C (2017). Gestational exposure to air pollution alters cortical volume, microglial morphology, and microglia-neuron interactions in a sex-specific manner. Front Synaptic Neurosci.

[53] Eggerstorfer B, Kim J-H, Cumming P, Lanzenberger R, Gryglewski G (2022). Meta-analysis of molecular imaging of translocator protein in major depression. Front Mol Neurosci.

[54] Andersson M, Tangen Ä, Farde L, Bölte S, Halldin C, Borg J (2021). Serotonin transporter availability in adults with autism – a positron emission tomography study. Mol Psychiatry.

[55] Mitelman SA, Buchsbaum MS, Young DS, Haznedar MM, Hollander E, Shihabuddin L (2018). Increased white matter metabolic rates in autism spectrum disorder and schizophrenia. Brain Imaging Behav.

[56] Chugani DC, Muzik O, Behen M, Rothermel R, Janisse JJ, Lee J (1999). Developmental changes in brain serotonin synthesis capacity in autistic and nonautistic children. Ann Neurol.

[57] Fung LK, Flores RE, Gu M, Sun KL, James D, Schuck RK (2021). Thalamic and prefrontal GABA concentrations but not GABA(A) receptor densities are altered in high-functioning adults with autism spectrum disorder. Mol Psychiatry.

[58] James D, Lam VT, Jo B, Fung LK (2022). Region-specific associations between gamma-aminobutyric acid A receptor binding and cortical thickness in high-functioning autistic adults. Autism Res.

[59] Coughlin JM, Wang Y, Ambinder EB, Ward RE, Minn I, Vranesic M (2016). In vivo markers of inflammatory response in recent-onset schizophrenia: a combined study using [11C]DPA-713 PET and analysis of CSF and plasma. Transl Psychiatry.

[60] Herranz E, Giannì C, Louapre C, Treaba CA, Govindarajan ST, Ouellette R (2016). Neuroinflammatory component of gray matter pathology in multiple sclerosis. Ann Neurol.

[61] Albrecht DS, Normandin MD, Shcherbinin S, Wooten DW, Schwarz AJ, Zurcher NR, et al. Pseudo-reference regions for glial imaging with 11C-PBR28: investigation in two clinical cohorts. J Nucl Med. 2018;59:107–114. 10.2967/jnumed.116.178335 PMC5750517 28818984

[62] Kreisl WC, Lyoo CH, Liow JS, Wei M, Snow J, Page E (2016). (11)C-PBR28 binding to translocator protein increases with progression of Alzheimer’s disease. Neurobiol Aging.

[63] Hooker JM, Carson RE (2019). Human positron emission tomography neuroimaging. Annu Rev Biomed Eng.

[64] Zurcher NR, Loggia ML, Lawson R, Chonde DB, Izquierdo-Garcia D, Yasek JE (2015). Increased in vivo glial activation in patients with amyotrophic lateral sclerosis: assessed with [(11)C]-PBR28. Neuroimage Clin.

[65] Lois C, Gonzalez I, Izquierdo-Garcia D, Zurcher NR, Wilkens P, Loggia ML (2018). Neuroinflammation in Huntington’s disease: new insights with (11)C-PBR28 PET/MRI. ACS Chem Neurosci.

[66] Kolb A, Wehrl HF, Hofmann M, Judenhofer MS, Eriksson L, Ladebeck R (2012). Technical performance evaluation of a human brain PET/MRI system. Eur Radiol.

[67] Bowen SL, Byars LG, Michel CJ, Chonde DB, Catana C (2013). Influence of the partial volume correction method on (18)F-fluorodeoxyglucose brain kinetic modelling from dynamic PET images reconstructed with resolution model based OSEM. Phys Med Biol.

