# Supplementary material for: In vivo translocator protein in females with autism spectrum disorder: a pilot study
Source: Neuropsychopharmacology. 2024 Apr 13;49(7):1193–201. doi: 10.1038/s41386-024-01859-6 (PMC11109261; doi:10.1038/s41386-024-01859-6)
Supplement: Supplementary file 1 — Supplement [file 41386_2024_1859_MOESM1_ESM.docx]

**Group comparison of [^11^C]PBR28 standardized uptake value normalized by whole brain mean (SUVR)**


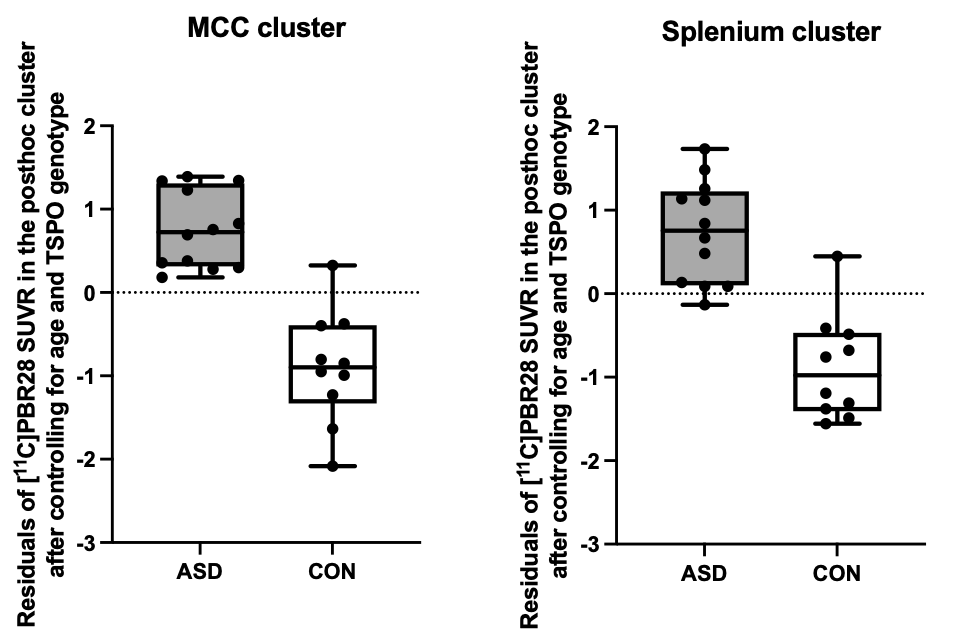


**Fig. S1.** Boxplot of the [^11^C]PBR28 SUVR residuals (controlling for age and TSPO genotype) in the posthoc clusters (MCC and splenium) where elevated [^11^C]PBR28 SUVR was found in ASD vs. CON females. Shown for visualization purposes. SUVR=standardized uptake value ratio, TSPO=translocator protein, MCC=midcingulate cortex.

**Structural volume**

To address potential confounds of volume, we assessed the structural volume of the posthoc clusters where group differences in [^11^C]PBR28 SUVR were found. Masks of the two posthoc clusters were transformed to each individual space and the structural volumes corresponding to the posthoc clusters were estimated for each individual. The structural volumes were normalized by each individual’s intracranial volume (ICV). The structural volume of the posthoc cluster/ICV was not different between ASD and CON groups in either cluster (MCC cluster: U=57, *p*=0.87, ASD median volume/ICV=1.14 x 10^-3^ [range: 0.92 x 10^-3^ – 1.55 x 10^-3^], CON median volume/ICV=1.11 x 10^-3^ [range: 0.92 x 10^-3^ – 1.28 x 10^-3^]; splenium cluster: U=44, *p*=0.31, ASD median volume/ICV=1.54 x 10^-3^ [range: 1.24 x 10^-3^ – 2.06 x 10^-3^], CON median volume/ICV=1.43 x 10^-3^ [range: 1.23 x 10^-3^ – 1.69 x 10^-3^]).

**Intellectual quotient (IQ) in ASD**

Given the range in IQ in the ASD group, in an exploratory analysis, this group was split into ASD with IQ<85 and ASD with IQ≥85 subgroups to test whether there were differences in [^11^C]PBR28 SUVR between these subgroups in the posthoc clusters. There was no difference in [^11^C]PBR28 SUVR in the subgroups of ASD (MCC cluster: ASD with IQ≥85 median SUVR=0.90 [range: 0.85-0.91], ASD with IQ<85 median SUVR=0.89 [range: 0.86-0.95]; splenium cluster: ASD with IQ≥85 median SUVR=0.82 [range: 0.77-0.88], ASD with IQ<85 median SUVR=0.83 [range: 0.73-0.89]) in either cluster, controlling for age and TSPO genotype (MCC cluster: U=14, p=0.64, splenium cluster: U=8, p=0.15, Fig. S2).

**
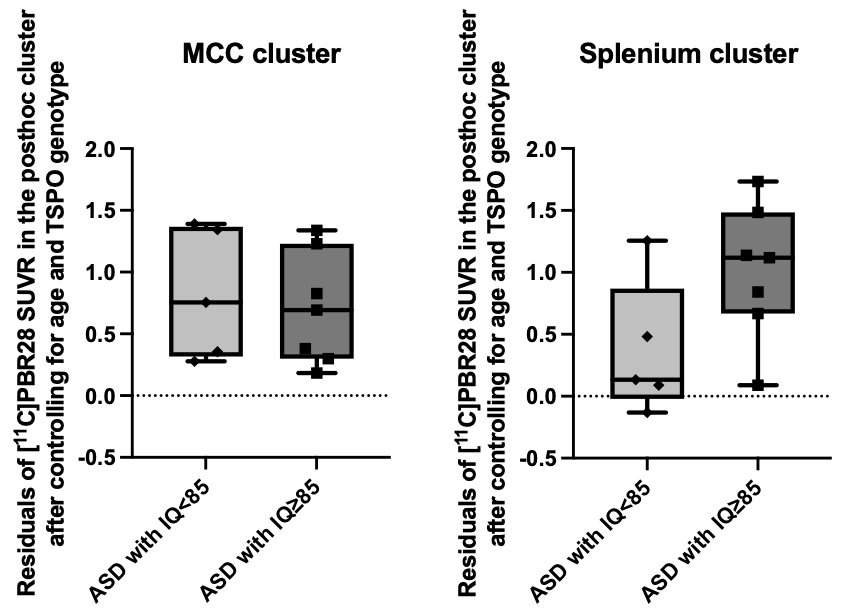
**

**Fig. S2.** Boxplot of the [^11^C]PBR28 SUVR residuals (controlling for age and TSPO genotype) in the posthoc clusters (MCC and splenium) where elevated [^11^C]PBR28 SUVR was found in ASD vs. CON females for the ASD subgroups (i.e., IQ≥85 and IQ<85). Shown for visualization purposes. SUVR=standardized uptake value ratio, TSPO=translocator protein, MCC=midcingulate cortex.

**Comorbid epilepsy**

We did not exclude for individuals with epilepsy as long as there was no seizure and no change in medication in the last 6 months. One participant with ASD had a history of epilepsy and analyzing the data without this subject did not significantly change the results. See Fig. S3.


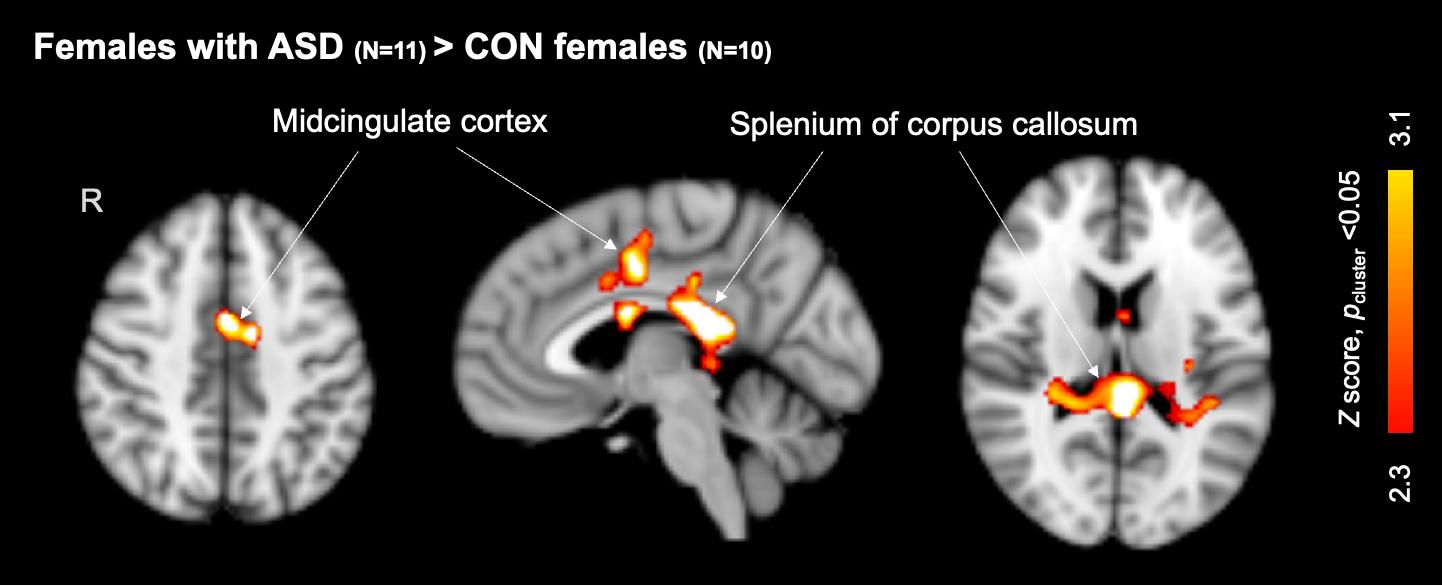


**Fig. S3.** Elevated *in vivo* [^11^C]PBR28 SUVR in female adults with ASD without comorbid epilepsy compared to matched CON. Statistical map from voxelwise comparison of [^11^C]PBR28 SUVR between groups, controlled for age and TSPO genotype, shows elevated regional TSPO levels relative to whole brain mean in the midcingulate cortex and splenium of the corpus callosum in ASD (N=11) compared to CON (N=10) (*Z*>2.3, *p_cluster_*<0.05). TSPO=translocator protein, SUVR=standardized uptake value ratio, N=number.

**Medication: benzodiazepines**

Lorazepam, desmethyldiazepam and oxazepam are benzodiazepines that show negligible binding affinity to TSPO and are thought to not affect radiotracer binding in TSPO PET studies. ^1^ We therefore did not exclude for these three medications, while we excluded for all other benzodiazepines. Among the study participants, one ASD participant was taking lorazepam. Analyzing the data without this subject did not significantly alter the results (Fig. S4).


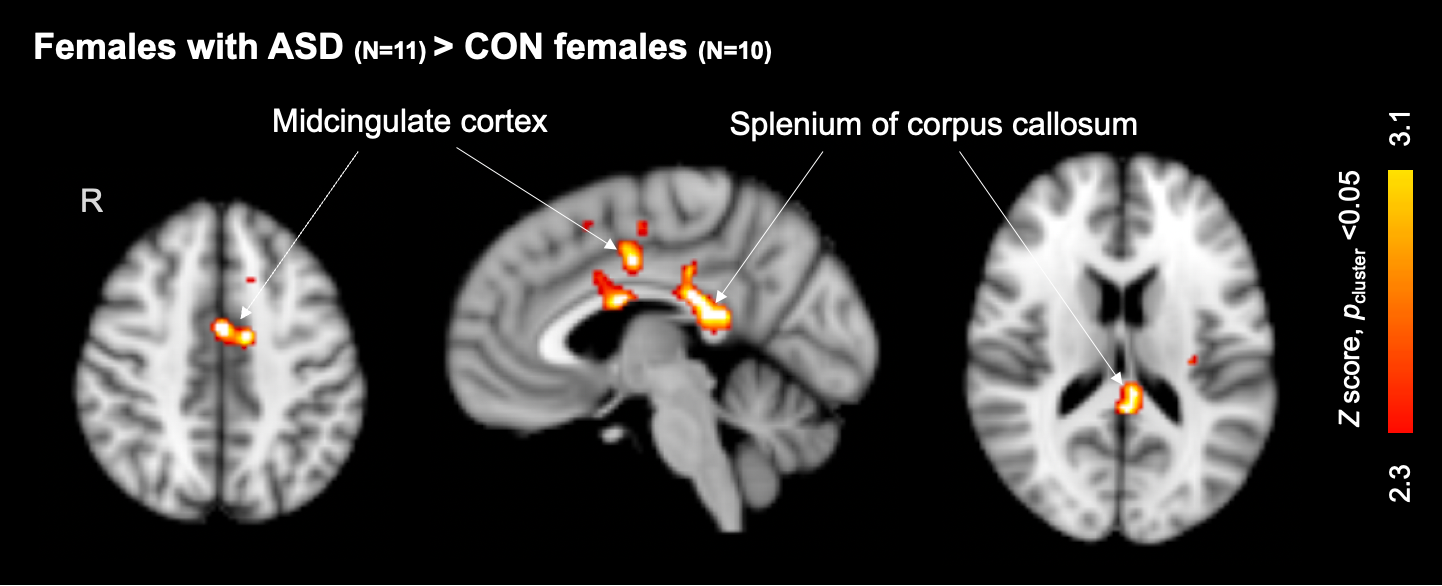


**Fig. S4.** Elevated *in vivo* [^11^C]PBR28 SUVR in female adults with ASD who were not taking benzodiazepines compared to matched CON. Statistical map from voxelwise comparison of [^11^C]PBR28 SUVR between groups, controlled for age and TSPO genotype, shows elevated regional TSPO levels relative to whole brain mean in the midcingulate cortex and splenium of the corpus callosum in ASD (N=11) compared to CON (N=10) (*Z*>2.3, *p_cluster_*<0.05). TSPO=translocator protein, SUVR=standardized uptake value ratio, N=number.

**Correlation between [^11^C]PBR28 SUVR and ADOS-2 total score**


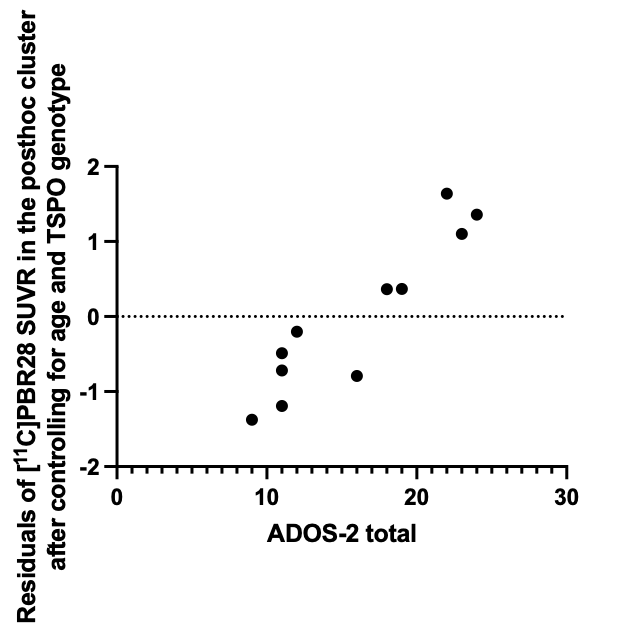


**Fig. S5.** Scatterplot of the [^11^C]PBR28 SUVR residuals (controlling for age and TSPO genotype) in the posthoc cluster where [^11^C]PBR28 SUVR was positively associated with ASD symptom severity as measured by ADOS-2 total score. Shown for visualization purposes. SUVR=standardized uptake value ratio, ADOS-2=Autism Diagnostic Observation Schedule-2.

**Self-report history of depression**

Exploratory analysis comparing [^11^C]PBR28 SUVR in the posthoc (ASD vs. CON) clusters between ASD who did and did not self-report a history of depression showed no differences between these subgroups (MCC cluster: ASD who self-reported a history of depression, median [^11^C]PBR28 SUVR=0.88 [range: 0.85-0.91]; ASD who did not self-report a history of depression, median [^11^C]PBR28 SUVR=0.90 [range: 0.86-0.95]; splenium cluster: ASD who self-reported a history of depression, median [^11^C]PBR28 SUVR=0.78 [range: 0.77-0.88]; ASD who did not self-report a history of depression, median [^11^C]PBR28 SUVR=0.83 [range: 0.73-0.89]), controlling for age and TSPO genotype (MCC cluster: U=15, p=0.76; splenium cluster: U=13, p=0.53).

Table S1

| **ROI** | **FRO** | **PAR** | **TEMP** | **OCC** | **INS** | **CING** | **CAU** | **PUT** | **PAL** | **THAL** | **HP_pHP** | **AMY** | **CB** | **WM** |
| --- | --- | --- | --- | --- | --- | --- | --- | --- | --- | --- | --- | --- | --- | --- |
| **ASD, scan 1** | 0.73±0.03 | 0.68±0.03 | 0.76±0.02 | 0.78±0.01 | 0.91±0.03 | 0.88±0.04 | 0.84±0.04 | 1.01±0.02 | 1.10±0.06 | 1.19± 0.08 | 1.00±0.04 | 1.08±0.03 | 0.90± 0.02 | 0.81±0.05 |
| **ASD, scan 2** | 0.73±0.01 | 0.67±0.02 | 0.78±0.03 | 0.78±0.03 | 0.91±0.03 | 0.85±0.02 | 0.83±0.04 | 1.00±0.02 | 1.07±0.05 | 1.19± 0.05 | 1.00±0.05 | 1.06±0.05 | 0.93±0.05 | 0.80±0.06 |
| **ASD, percent signal change** | -0.11± 2.63 | -0.84±3.38 | 2.09±1.86 | 0.07±3.70 | -0.09±0.84 | -2.74±5.53 | -1.29±2.35 | -1.43±1.08 | -2.39±3.39 | -0.33±3.41 | -0.22±3.37 | -2.00±1.75 | 2.56±5.35 | -1.86±1.61 |
| **CON, scan 1** | 0.72±0.03 | 0.66±0.05 | 0.76±0.03 | 0.79±0.05 | 0.92±0.05 | 0.84±0.02 | 0.78±0.04 | 0.99±0.04 | 1.03±0.05 | 1.14±0.04 | 0.95±0.03 | 1.01±0.04 | 0.93±0.11 | 0.79±0.09 |
| **CON, scan 2** | 0.73±0.02 | 0.67±0.05 | 0.76±0.02 | 0.78±0.06 | 0.91±0.04 | 0.84±0.04 | 0.77±0.03 | 0.98±0.03 | 1.05±0.04 | 1.11±0.04 | 0.94±0.02 | 1.01±0.06 | 0.94±0.09 | 0.78±0.09 |
| **CON, percent signal change** | 0.87±1.66 | -0.06±1.21 | 0.10±1.33 | -1.76±1.76 | -0.53±2.71 | -0.10±2.44 | -1.18±3.40 | -0.58±2.12 | 2.04±3.20 | -2.01±4.60 | -0.86±2.04 | -0.46±3.99 | 0.84±2.89 | -1.43±2.10 |

[^11^C]PBR28 standardized uptake value normalized by whole brain mean for scan 1 and scan 2 and percent signal change between the two time points across subjects that had two scans (ASD N=4, CON N=5). All values are mean ± standard deviation. ROI=region of interest, FRO=frontal, PAR=parietal, TEMP=temporal, OCC=occipital, INS=insula, CING=cingulate, CAU=caudate, PUT=putamen, PAL=pallidum, THAL=thalamus, HP_pHP=hippocampus/parahippocampal gyrus, AMY=amygdala, CB=cerebellum, WM=white matter.

**Reference**

1. Kalk NJ, Owen DR, Tyacke RJ, et al. Are prescribed benzodiazepines likely to affect the availability of the 18 kDa translocator protein (TSPO) in PET studies? Synapse 2013;67:909-912.
